# Info-Telefon Depression – eine explorative Analyse von Anruferdaten eines bundesweiten Informationsangebots zu depressiven Erkrankungen

**DOI:** 10.1007/s00115-020-00921-0

**Published:** 2020-05-13

**Authors:** Frauke Görges, Christian Gravert, Christine Rummel-Kluge, Ulrich Hegerl

**Affiliations:** 1 Klinik und Poliklinik für Psychiatrie und Psychotherapie, Universitätsklinikum Leipzig, Semmelweisstraße 10, Haus 13, 04103 Leipzig, Deutschland; 2 Stiftung Deutsche Depressionshilfe, Leipzig, Deutschland; 3 Deutsche Bahn Stiftung gGmbH, Berlin, Deutschland; 4 Klinik für Psychiatrie, Psychosomatik und Psychotherapie, Goethe-Universität Frankfurt, Frankfurt, Deutschland

Depression ist mit einer 12-Monats-Prävalenz von 8,2 % eine der häufigsten psychischen Erkrankungen in Deutschland [1]. Obwohl evidenzbasierte, wirksame Behandlungsmethoden zur Verfügung stehen, finden nach wie vor viele Betroffene den Weg in die Versorgung nicht [2]. Bis ein Betroffener aktiv nach professioneller Unterstützung sucht, werden nach Schomerus et al. [3] verschiedene Stufen durchlaufen: die Selbstidentifikation als psychisch erkrankt, die Wahrnehmung der Notwendigkeit zur Behandlung, die Intention, sich Hilfe zu suchen, und zuletzt das eigentliche Hilfesuchverhalten. Diese Stufen können durch verschiedene Faktoren, wie die Einstellungen des Betroffenen oder das Wissen über die Erkrankung, beeinflusst werden.

Tomczyk et al. [4] zeigten in ihrer Studie, dass das Wissen um lokale Versorgungsangebote die vierte Stufe, das tatsächliche Hilfesuchverhalten der Betroffenen, positiv beeinflussen kann. In ihrer Studie gab jedoch lediglich um die Hälfte der Befragten an, zu wissen, wo sie einen professionellen Behandler für psychische Erkrankungen finden können. Andere Studien zeigen, dass in der Bevölkerung weiterhin viele Vorurteile und Wissenslücken zum Thema Depression und ihrer Behandlung existieren [5, 6]. Informationen können zwar leicht im Internet recherchiert werden, sind für Laien jedoch häufig nur schwer auf ihre Richtigkeit zu prüfen.

Der Bedarf an leicht zugänglichen, verlässlichen, qualitätsgesicherten Informationen zur Erkrankung Depression, zu deren Behandlungsmöglichkeiten, zum Versorgungssystem und auch zum Umgang mit Betroffenen ist demnach groß, was sich bereits vor der Etablierung des Info-Telefons Depression in der hohen Anzahl von Anfragen an die Stiftung Deutsche Depressionshilfe widerspiegelte.

## Das Info-Telefon Depression

Basierend auf den positiven Erfahrungen anderer telefonischer Informationsangebote aus dem somatischen und dem psychischen Bereich, wie dem Krebsinformationsdienst [7] oder der Informations-Hotline des Kompetenznetzes Schizophrenie [8], wurde im Jahr 2014 in Kooperation mit der Deutsche Bahn Stiftung das Info-Telefon Depression ins Leben gerufen.

Das Ziel des Info-Telefons Depression ist es, Betroffenen, Angehörigen und anderen Interessenten niederschwellig qualitätsgesicherte Informationen rund um das Thema Depression zur Verfügung zu stellen. Individuelle Beratung sowie therapeutische und Kriseninterventionen stehen hingegen nicht im Fokus und werden von anderen Einrichtungen abgedeckt. Das Info-Telefon ist bundesweit an 5 Tagen in der Woche für jeweils 4 h unter der kostenfreien Rufnummer 0800 33 44 5 33 erreichbar und wird von Psychologen betreut.

## Evaluation

In den ersten Jahren nach Etablierung des Info-Telefons Depression wurden im Rahmen einer explorativen Studie demographische, zeitliche und inhaltliche Daten erhoben. Ziel war es, den Informationsbedarf zur Erkrankung, zu Behandlungsmöglichkeiten und zum Versorgungssystem systematisch zu erfassen und so die Grundlage für eine verbesserte, zielgruppenorientierte und spezifische Informationsvermittlung durch die Stiftung Deutsche Depressionshilfe sowie andere Institutionen und Gatekeeper zu schaffen. Hierzu wurden die entsprechenden Angaben, wie Anrufertyp, Anrufgrund oder Gesprächsdauer, durch Mitarbeiter des Info-Telefons Depression erfasst und fehlende Angaben, wie Geschlecht oder Alter, zusätzlich von Anrufern erfragt. Durch sofortige Kategorisierung der Angaben bei der Datenspeicherung wurde die Anonymisierung der Daten gewährleistet. Den Info-Telefon-Mitarbeitern wurde hierfür eine Datei mit den zu erhebenden Merkmalen sowie durch Drop-down-Menüs vorgegebene Antwortkategorien zur Verfügung gestellt, die zuvor von Stiftungsmitarbeitern anhand früherer Erfahrungen erarbeitet worden war. Ein positives Ethikvotum der Ethikkommission der Universitätsmedizin Leipzig liegt vor.

Ausgeschlossen wurden Daten von Anrufern, die eine Erhebung ihrer Angaben explizit ablehnten sowie Daten Minderjähriger. Ausgewertet wurden die Daten aller weiteren Anrufer im Zeitraum April 2015 bis März 2019, mit denen ein Gespräch zustande kam. Insgesamt wurden Daten von 8232 Anrufern in die Auswertung einbezogen. Es wurden auch Anrufer in die Auswertung einbezogen, bei denen nicht alle relevanten Daten erfasst werden konnten. So kann es beispielsweise vorkommen, dass Info-Telefon-Mitarbeiter bei hoch emotionalen Gesprächen entscheiden, fehlende Angaben der Anrufer, wie z. B. das Alter oder das Medium, über welches der Anrufer auf das Angebot aufmerksam geworden war, nicht nachzuexplorieren.

## Ergebnisse der Evaluation

Das Angebot wurde überwiegend von Frauen und jüngeren Menschen unter 50 Jahren genutzt. Über etwas mehr als die Hälfte der Anrufe wurde von Angehörigen getätigt, gefolgt von Betroffenen und, selten, sonstigen Interessenten (siehe Tab. 1 für eine Ergebniszusammenfassung).

**Table 1:** 

Merkmal	(*n*)	(%)
*Geschlecht*		
Weiblich	5331	65,1
Männlich	2862	34,9
*Alter (Jahre)*		
<50	4219	62,0
≥50	2589	38,0
*Anrufertyp*		
Angehöriger	4318	52,8
Betroffener	3655	44,7
Sonstiger	207	2,5
*Jahreszeit des Anrufs*		
Frühling	1886	22,9
Sommer	1919	23,3
Herbst	2219	27,0
Winter	2208	26,8
*Gesprächsdauer*		
<5 min	1231	15,1
5–10 min	2730	33,4
10–15 min	2151	26,3
>15 min	2058	25,2

Angegeben werden gültige Prozente

Während die anrufenden Angehörigen zu 69,2 % weiblich waren, waren die Betroffenen, auf die sich diese Anrufe bezogen, zu 52,7 % männlich. Ein Großteil der Betroffenen hatte vor der Kontaktaufnahme mit dem Info-Telefon bereits eine Depressionsdiagnose erhalten (57,9 %) und war bereits vor über einem Jahr erstmalig erkrankt (62,6 %).

Der häufigste Anrufgrund unter Angehörigen war die Frage nach dem richtigen Umgang mit betroffenen Verwandten oder Freunden, gefolgt von der Suche nach allgemeinen Informationen zum Thema Depression sowie Fragen zum Versorgungssystem. Unter betroffenen Anrufern wurden hingegen am häufigsten allgemeine Informationen zum Thema Depression gesucht, gefolgt vom Wunsch nach einem therapeutischen Gespräch und Fragen zum Versorgungssystem sowie der Informationssuche bei bisheriger erfolgloser Behandlung bzw. Unzufriedenheit mit dieser (Tab. 2).

**Table 2:** 

Anrufgründe	*n*	%
*Angehörige*		
Umgang mit betroffenen Angehörigen	2930	32,4
Suche nach allgemeinen Informationen zu Depression	956	10,6
Fragen zum Versorgungssystem	726	8,0
Weitere Anrufgründe	4430	49,0
*Betroffene*		
Suche nach allgemeinen Informationen zu Depression	920	12,9
Wunsch nach therapeutischem Gespräch	781	11,0
Fragen zum Versorgungssystem	718	10,1
Informationssuche bei erfolgloser Behandlung/Unzufriedenheit	717	10,1
Weitere Anrufgründe	3969	55,9

Angabe von bis zu vier Anrufgründen pro Anrufer möglich; angegeben wird der Anteil an Anrufgründen innerhalb des jeweiligen Anrufertyps; weitere Anrufgründe beinhalten: spezifische Informationssuche, Suche nach Klinik/Therapeut, rechtliche Fragen, Fragen zu Kostenträgern, Diagnosewunsch, Informationssuche zu Psychotherapie, Medikamenten, spezifischen biologischen und sonstigen Behandlungsangeboten, Suche nach Beratungsstellen, Hilfsangeboten für Angehörige, Selbsthilfegruppen oder Austausch mit anderen Betroffenen/Angehörigen

Der Großteil der Anrufer gab an, das Angebot zum ersten Mal zu nutzen (93,7 %) und über das Internet auf das Info-Telefon aufmerksam geworden zu sein (79,8 %). Die restlichen Anrufer gaben an, über Zeitungen (7,3 %), Freunde und Bekannte (4,0 %), das Fernsehen (3,0 %) sowie professionelle Helfer, Plakate und Flyer, das Radio, Bücher, den Stiftungsnewsletter oder sonstige Informationsquellen (insgesamt 4,9 %) auf das Angebot aufmerksam geworden zu sein. Unter den jüngeren Anrufern gaben 88,2 % an, über das Internet auf das Info-Telefon Depression aufmerksam geworden zu sein. Unter den älteren Anrufern lag diese Zahl bei lediglich 64,9 % (χ^2^[1, *n* = 6363] = 493,8, *p* < 0,001). Das Angebot wurde von Jahr zu Jahr stärker genutzt (1. Jahr: 1337 Anrufe, 4. Jahr: 3175 Anrufe). Im Herbst und Winter gab es etwas mehr Anrufe als im Frühling und im Sommer. Die Gesprächsdauer bewegte sich bei den meisten Anrufern zwischen 5 und 10 min.

## Diskussion

Das Info-Telefon Depression wurde in den ersten vier Jahren gut angenommen und die Nachfrage wuchs stetig. Bereits in den ersten zwei Jahren wurden fast 5‑mal so viele Anrufe realisiert wie bei der vergleichbaren Hotline des Kompetenznetzes Schizophrenie. Ähnlich wie bereits bei der Schizophrenie-Hotline wurde das Info-Telefon Depression zu einem großen Anteil von informationssuchenden Angehörigen genutzt [8]. Wie z. B. auch bei der Telefonseelsorge wurden ca. zwei Drittel der Anrufe von Frauen getätigt. Während jedoch der Anteil der unter 50-Jährigen bei der Telefonseelsorge weniger als die Hälfte der Anrufe ausmachte, lag der Anteil beim Info-Telefon Depression bei fast zwei Dritteln [9]. Dieser Unterschied könnte auf das Ausweichen jüngerer Personen auf schriftliche Kommunikation, z. B. über den Chat, zurückzuführen sein. Der im Vergleich zum Bevölkerungsdurchschnitt jüngere Anteil von Anrufern beim Info-Telefon Depression könnte auf die Bekanntmachung des Angebots im Internet zurückzuführen sein, welches vermehrt von dieser Altersgruppe genutzt werden dürfte. Weitere Punkte, die darauf hindeuten, dass sich Nutzergruppen und damit die Natur der Anrufe zwischen Informationstelefonen und Sorgen‑/Krisentelefonen unterscheiden, sind die Anrufdauer, die z. B. bei der Telefonseelsorge bedeutend höher zu sein scheint (häufigste Kategorie: 15–30 min) als beim Info-Telefon Depression (häufigste Kategorie: 5–10 min) oder der Schizophrenie-Hotline (durchschnittliche Gesprächsdauer: 8,5 min), sowie der Anteil von Anrufern, die zum wiederholten Mal anrufen. Während dieser Anteil bei beiden Informationstelefonen unter 10 % lag, war er bei der Telefonseelsorge etwa 5‑mal so hoch wie der Anteil derer, die zum ersten Mal anriefen [8, 9].

Limitationen der Erhebung liegen vor allem in der Notwendigkeit, die Datenerhebung möglichst kurz zu halten und erhobene Daten für die Gewährleistung der Anonymität stark zu kategorisieren, wodurch eine Fokussierung auf die relevantesten Aspekte und die Auswertung auf einem relativ hohen Abstraktionsniveau notwendig wurden. Weiterhin stand für einen Teil der Daten, wie beispielsweise die Auskunft über das Vorliegen einer professionellen Depressionsdiagnose, lediglich die Selbstauskunft der Anrufenden ohne Maße zur Objektivierung zur Verfügung.

## Ausblick

Das Info-Telefon Depression stellt ein Angebot vor allem für solche Anrufer zur Verfügung, die auf der Suche nach wissenschaftlich fundierten Informationen rund um die Erkrankung Depression sind. Die stetig wachsende Nachfrage spricht dafür, das Projekt auch in Zukunft weiterzuführen. Ein wichtiger Punkt sollte dabei die Evaluation der Zufriedenheit der Anrufer sein.

## Fazit für die Praxis

- Bereits in den S3-Leitlinien zur unipolaren Depression wird der Einbezug Angehöriger empfohlen. Dem großen Informationsbedarf Angehöriger zum Umgang mit Betroffenen sollte mit entsprechenden Angeboten Rechnung getragen werden. Wissenschaftlich fundierte Angebote für Angehörige, wie der „Familiencoach Depression“, stehen bereits zur Verfügung.
- Viele betroffene Anrufer sind bereits in Behandlung; Informationsmaterial in Praxen zum Thema Depression, zu (neueren) Behandlungsansätzen sowie zum Versorgungssystem kann Informationslücken schließen. Wissenschaftlich fundiert und leicht zugänglich ist die „PatientenLeitlinie zur Nationalen VersorgungsLeitlinie Unipolare Depression“.
- Bei leichteren Depressionsformen können, in Anlehnung an die Empfehlung der S3-Leitlinie zum Einsatz niederschwelliger Interventionen, beispielsweise Internetinterventionen wie „iFightDepression“ oder „MoodGym“ – beide wissenschaftlich fundiert und frei zugänglich – vorgeschlagen werden.

## References

[1] Jacobi F, Höfler M, Strehle J (2016). Erratum zu: Psychische Störungen in der Allgemeinbevölkerung. Studie zur Gesundheit Erwachsener in Deutschland und ihr Zusatzmodul „Psychische Gesundheit“ (DEGS1-MH) (Erratum to: Mental disorders in the general population. Study on the health of adults in Germany and the additional module mental health (DEGS1-MH)). Nervenarzt.

[2] Mack S, Jacobi F, Gerschler A (2014). Self-reported utilization of mental health services in the adult German population—evidence for unmet needs? Results of the DEGS1-Mental Health Module (DEGS1-MH). Int J Methods Psychiatr Res.

[3] Schomerus G, Stolzenburg S, Freitag S (2019). Stigma as a barrier to recognizing personal mental illness and seeking help: a prospective study among untreated persons with mental illness. Eur Arch Psychiatry Clin Neurosci.

[4] Tomczyk S, Schmidt S, Muehlan H (2019). A prospective study on structural and attitudinal barriers to professional help-seeking for currently untreated mental health problems in the community. J Behav Health Serv Res.

[5] Althaus D, Stefanek J, Hasford J (2002). Wissensstand und Einstellungen der Allgemeinbevölkerung zu Symptomen, Ursachen und Behandlungsmöglichkeiten depressiver Erkrankungen. Nervenarzt.

[6] Freitag S, Stolzenburg S, Schomerus G (2018). Depressionswissen – Deutsche Übersetzung und Testung der Depression Literacy Scale (Depression Literacy—German Translation and Testing of the Depression Literacy Scale). Psychiatr Prax.

[7] Weg-Remers S (2014). Der Krebsinformationsdienst des Deutschen Krebsforschungszentrums (The cancer information service of the German Cancer Research Center). Radiologe.

[8] Wessling A, Wölwer W, Heres S (2006). Telefon-Hotline als niederschwelliges Angebot für Fragen zur Schizophrenie (A telephone hotline as an easily accessible service for questions on schizophrenia). Nervenarzt.

[9] https://www.telefonseelsorge.de/sites/default/files/Statistik_ts_in_deutschland_2017_0.pdf. Zugegriffen: 20. Dez. 2019

[10] DGPPN, BÄK, KBV, AWMF (2015). S3-Leitlinie/Nationale VersorgungsLeitlinie Unipolare Depression – Langfassung.

[11] https://depression.aok.de/zum-familiencoach-depression. Zugegriffen: 20. Dez. 2019

[12] https://www.patienten-information.de/mdb/downloads/nvl/depression/depression-2aufl-vers2-pll.pdf. Zugegriffen: 20. Dez. 2019

[13] https://ifightdepression.com/de/selbstmanagement-ressourcen/ifightdepression-tool. Zugegriffen: 20. Dez. 2019

[14] https://moodgym.de. Zugegriffen: 20. Dez. 2019

